# Enhancing Privacy Controls for Patients via a Selective Authentic Electronic Health Record Exchange Service: Qualitative Study of Perspectives by Medical Professionals and Patients

**DOI:** 10.2196/10954

**Published:** 2018-12-21

**Authors:** Ala Sarah Alaqra, Simone Fischer-Hübner, Erik Framner

**Affiliations:** 1 Privacy and Security Research Group Department of Computer Science Karlstad University Karlstad Sweden; 2 Department of Information Systems Karlstad University Karlstad Sweden

**Keywords:** privacy, patient data privacy, electronic health record, user control, data protection, data security, eHealth, human computer interaction

## Abstract

**Background:**

Patients’ privacy is regarded as essential for the patient-doctor relationship. One example of a privacy-enhancing technology for user-controlled data minimization on content level is a redactable signature. It enables users to redact personal information from signed documents while preserving the validity of the signature, and thus the authenticity of the document. In this study, we present end users’ evaluations of a Cloud-based selective authentic electronic health record (EHR) exchange service (SAE-service) in an electronic health use case. In the use case scenario, patients were given control to redact specified information fields in their EHR, which were signed by their doctors with a redactable signature and transferred to them into a Cloud platform. They can then selectively disclose the remaining information in the EHR, which still bears the valid digital signature, to third parties of their choice.

**Objective:**

This study aimed to explore the perceptions, attitudes, and mental models concerning the SAE-service of 2 user roles: signers (medical professionals) and redactors (patients with different technical knowledge) in Germany and Sweden. Another objective was to elicit usability requirements for this service based on the analysis of our investigation.

**Methods:**

We chose empirical qualitative methods to address our research objective. Designs of mock-ups for the service were used as part of our user-centered design approach in our studies with test participants from Germany and Sweden. A total of 13 individual walk-throughs or interviews were conducted with medical staff to investigate the EHR signers’ perspectives. Moreover, 5 group walk-throughs in focus groups sessions with (N=32) prospective patients with different technical knowledge to investigate redactor’s perspective of EHR data redaction control were used.

**Results:**

We found that our study participants had correct mental models with regard to the redaction process. Users with some technical models lacked trust in the validity of the doctor’s signature on the redacted documents. Main results to be considered are the requirements concerning the accountability of the patients’ redactions and the design of redaction templates for guidance and control.

**Conclusions:**

For the SAE-service to be means for enhancing patient control and privacy, the diverse usability and trust factors of different user groups should be considered.

## Introduction

### Background

Privacy has been acknowledged by the Council of Europe’s Convention on Human Rights in 1950 as a basic human right. A well-acknowledged definition of privacy was provided by the German Constitutional Court, which defined privacy as the right to informational self-determination [1], allowing individuals to determine for themselves (and thereby control) what personal information about themselves they disclose under which conditions to others.

Due to the sensitivity of medical data, the privacy of patients has been seen as essential for trust relationship between medical professionals and patients [2] over centuries, as addressed by the Hippocratic Oath [2,3].

One fundamental privacy principle that entails control over information is data minimization. It states that privacy can be best protected if personal data are not collected nor processed at all or if the amount of personal data processing is limited to the minimum necessary, at least. As the European Union (EU) General Data Protection Regulation (GDPR) requires in its Art 5 I (c), personal data shall be “adequate, relevant and limited to what is necessary in relation to the purposes for which they are processed” (data minimization) [4].

Broad ranges of Privacy Enhancing Technologies (PETs) have been developed for technically enforcing data minimization that play a key role when designing systems for privacy. One example of such a PET for data minimization on content level is redactable signatures (also called malleable signatures), which enable the redaction (*blacking-out*) of personal information from signed documents while preserving the validity of the signatures [5].

In the EU H2020 project PRivacy and Security MAintaining services in the CLOUD (PRISMACLOUD), redactable signatures are used for developing a Cloud-based selective authentic electronic health record (EHR) exchange service (SAE-service) in a privacy-enhanced electronic health (eHealth) use case. In contrast to traditional digital signatures, which imply that any changes to a signed document will invalidate the signature, redactable signatures allow the controlled redaction of certain parts of the signed data without the signature losing its validity. Any unauthorized modification would, however, invalidate the signature. Hence, both authenticity and integrity of the data are protected.

An EHR is defined as “computerized record of a person’s health and/or medical history...’’ [6-8]. In our studies, we have considered the EHR term in the hospital system for referring to medical documents. However, some might consider signed EHRs in the Cloud portal of our scenario to be personal health records (PHRs). As the concept of PHR has been noted by Wiljer et al [8] to be controversial, and was stated that no widely accepted definition exists, we, therefore, refrained from using the term PHR in our study. In addition, in our scenario, medical documents are to be used for medical purposes (second diagnosis). For simplicity reasons, we chose to use the terms EHR and medical document or record interchangeably in this paper for both the hospital and the Cloud portal.

**Figure 1:**
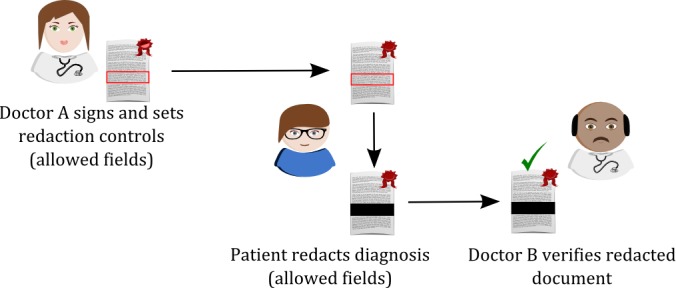
Redactable signatures in the PRISMACLOUD (PRivacy and Security MAintaining services in the CLOUD) eHealth use-case.

In the PRISMACLOUD eHealth use case, patients are given control and allowed to redact information in their EHR (Figure 1). In a hospital system, a medical professional (doctor A) signs the EHR with a redactable signature. The EHR is then transferred to the patient’s account on a hospital Cloud platform. The patient is then able to *black-out* the predefined redactable fields of information from the signed EHR copy on the Cloud portal. Meanwhile, the signature of doctor A remains valid and the authenticity of the medical document is maintained as long as the patient is following the redaction rules. For instance, if the patient wants to get a second opinion on a diagnosis of their EHR containing blood test results, the diagnosis fields could be redacted from the EHR by the patient. The redacted EHR including only the blood values is then made available on the Cloud portal to a specialist of the patient’s choice. The specialist (doctor B) can validate the signature by doctor A, and thus, verify the authenticity of the patient’s blood value data (that they are indeed medical data that were collected by doctor A), which is important for protecting the patient’s safety.

Hence, both user-controlled data minimization and authenticity of the selectively disclosed medical data can be provided. In an alternative use case, for producing a signed sick leave letter for the employer, the patient could redact all fields except for the fields stating the period for that the patient stayed in the hospital.

Redactions can be either implemented as an unkeyed operation that allows any party to redact the document or as a keyed operation requiring that the redactor uses a secret redaction key, which means that the redactor could later also be made accountable for the redaction.

A recent Eurobarometer survey requested by the European Commission showed that a majority of respondents would like Web-based access to their medical records, whereas the question whether they would like to grant access to their records to third parties depends on the type of recipient [9]. Moreover, earlier studies revealed that patient- (or more generally, user-) determined privacy controls and restrictions on the content and/or recipient may be a prerequisite of sharing [10,11], whereas privacy concerns and a lack of selective controls have a negative influence on the intention to share medical information even with other health care providers [12] and may reduce patient care quality [13]. As discussed in Caine et al’s study [14], patients would like to have granular privacy controls over their health information in medical records allowing them to differentially share their data in medical records or only parts of it, depending on the data recipient of and/or type of medical data. The SAE-service provides a technical solution for such granular privacy control that is demanded by Caine et al [14] for maintaining the level of privacy afforded by medical records and for achieving alignment with patient preferences. At the same time, the SAE-service also protects the authenticity of the selectively disclosed data for safeguarding the patient’s safety.

From a human-computer interaction (HCI) standpoint, the design of user interfaces (UIs) for such SAE-service poses several challenges. In particular, privacy crypto schemes may be counter-intuitive to users. Therefore, it is a challenge to design UIs for evoking comprehensive mental models [15]. This problem is increased by the fact that redactable signatures work differently from traditional signature schemes, which in contrast to redactable signatures, get invalid if the signed document is redacted, that is, changed. This may affect the trust that users with some familiarity with crypto technologies may have in such a PET. Moreover, different user groups among medical professionals and patients may have different expectations and requirements concerning this SAE-service, which need to be appropriately addressed.

The importance of end users’ participation as stakeholders in the privacy by design (PbD) process, involving multiple disciplines, including usability design in addition to engineering, has been emphasized earlier [16]. End users should ultimately profit from PbD, where it has been pointed out that UIs need to address PbD and be “human-centered, user-centric, and user-friendly, so that informed privacy decision may be reliably exercised” [17]. Throughout the PRISMACLOUD project, we have followed the user-centered design (UCD) approach [18], which meant that the focus was on users throughout the development, design, and evaluation of UI prototypes for this SAE-service. The eHealth use case addressed 2 types of stakeholders who were involved as end users in our studies. They are the signers of medical documents who are medical professionals (eg, doctors) and redactors of medical documents who are users playing the role of patients.

### Objective

This study reports about the results of our research that has been addressing the following research questions:

- What are the perceptions, attitudes, and mental models that users of both roles, signers (medical professionals) and redactors (patients with different technical knowledge), from Germany and Sweden, have with regard to patient-controlled redactions as part of the SAE-service?

- What are end user requirements for making of redactable signatures as part of the SAE-service usable?

We have included individuals with varying technical background performing the redactor’s role, as we were interested in investigating whether different levels of knowledge of crypto technologies will affect their understanding and trust in redactable signatures. Moreover, as this study is conducted within the scope of an EU research project, we involved end users in 2 EU countries (corresponding to project partner’s locations: Germany and Sweden), which also allowed us to investigate possible national influences. The first research question looks into the broader contribution of understanding users and serves as a prerequisite to the second research question where requirements are derived for the SAE-service.

## Methods

### Overview

We have followed a UCD approach for developing and evaluating UI prototypes for this SAE-service. UCD approach focuses on the needs of users and integrating that into the design processes [18].

Therefore, in our previous work, we involved end users for the elicitation of an initial set of requirements (found in Table 1). It was first done via semistructured interviews and stakeholder workshops as described and analyzed in Alaqra et al’s study [19].

These initial requirements were considered for the design of low-fidelity UI prototypes (mock-ups) for the SAE-service (as shown in the subsection Mock-Ups User Interface Design). The design of the mock-ups went through several iterations of experts’ reviews and walkthroughs, which were used in this study for the evaluation and facilitation of the discussion of both of our studies (shown in the following sections).

As our research is explorative with the objective to investigate and gain a deeper understanding about the users’ perceptions, attitudes, and mental models, we chose qualitative empirical means in our approach.

In total, there were 2 studies conducted with the 2 categories of users, that is, the signers and the redactors, respectively. Both studies followed a semistructured format, unlike structured methods, which allows the freedom and openness of the discussions to explore one’s perspectives and opinions. However, both studies had a specific set of topics for the discussions. These topics are presented and highlighted in the mock-ups UI themes, which served as the main facilitator of the discussions (see section Mock-ups User Interface Design for Electronic Health).

The 2 studies conducted were corresponding to the mock-ups parts: (1) individual walk-throughs (interviews) with medical professionals to evaluate the hospital platform mock-ups and (2) group walk-throughs (focus groups; FGs) with users, prospective patients, to evaluate the Cloud portal mock-ups.

### Recruitment

Both studies were conducted with participants in Germany and Sweden (specifically Värmland County), not only because the PRISMACLOUD consortium includes partners from those countries but also because they are different in terms of the digitization of eHealth infrastructures. Sweden is regarded as one of leading EU countries in eHealth use [20,21] and national EHR system development [22]. The significant progress in moving toward eHealth has been contributed by a well-developed Information and Communication Technology (ICT) infrastructure [20], with a fully integrated EHR system on both county and national level.

In accordance with the national patient summary *Nationell patientöversikt* strategy, health care professionals can be given direct access to a patient’s health records that are kept by a care provider in any of the country’s 21 county councils. Via the national Web-based portal, citizens in a number of counties, including Värmland, have access to view their personal health data and request services [22]. In Germany, on the other hand, patient data are mainly documented on paper; and previous studies show that Germany is facing multiple obstacles that prevent the implementation of a national EHR system [20,22,23]. Moreover, as also the latest Eurobarometer survey on data protection from 2015 confirmed, citizens in those 2 countries have different levels of perceptions with regard to control over their personal sphere on the Web and trust that individuals have in different entities: Germany had, for instance, the highest number of respondents who think that they have no control or only partial control over their personal data, whereas people in Sweden are most likely to trust their national public authorities [24]. All these different factors motivated our choice of conducting our studies in both Germany and Sweden for also analyzing possible national influences.

For both studies, we invited participants via professional and personal contact networks and offered them lunch as a compensation. All interviews and FGs took place between mid-May and mid-June 2017.

### Documentation and Analysis

For every FG and interview, there were 2 expert interviewers: 1 moderator for the discussion and 1 for note taking. Voice recording was used as a reference for the notetaking process. After the sessions, the interviewers documented their notes using the voice recording. Notes were collected and combined from the interviewers, then were iteratively categorized and evaluated into themes. Finally, the initial requirements were refined and concluded under each theme, which are summarized and presented in the Results section.

### Ethical Review

Our evaluation research plan was submitted to the ethics review board at Karlstad University for approval. They decided in their meeting on May 9, 2017 that our evaluation experiment would not fall under the Swedish Ethical Review Act [25] and were, therefore, approved before we started with conducting our evaluations.

Participation in the interviews and FGs was restricted to adult volunteers, who provided their consent after being informed, both orally and in written form, about our privacy policy.

According to the Swedish Ethical Review Act, ethical review by a regional ethical review board would be required if sensitive personal data were collected or processed within the scope of the research project. We conducted FGs with users in the role of a patient; however, did not collect any of their personal medical data. We clearly advised all participants to take the role of a specified persona, that is, of made-up persons, during the FG discussions. We strictly advised them to not talk about any personal matters and confine their discussions to their persona’s point of view. We informed them that in case they talked about any personal sensitive information, we would stop the recording of the session directly and delete that recorded part.

### Individual Walk-Throughs: Interviews

These interviews were conducted to understand medical professionals’ perspectives and opinions regarding redactable signatures from the signer’s point of view. Currently, in the given eHealth scenario, doctors will have to sign the EHR with redactable signatures. We chose individual walk-throughs, that is, one-on-one interviews, as medical professionals who were recruited came from different fields and had different expertise. In addition, it was technically not plausible to gather many doctors at a specific time to conduct an evaluation.

### Protocol

For addressing the signers’ of redactable signatures perspectives, we used the hospital platform mock-ups. Individual walk-throughs were carried out with medical staff in the form of semistructured interviews that lasted an average of 35 to 40 min. Consent forms were explained and handed out for participating in the study and for recording the session (see Multimedia Appendix 1. Consent form for interview participants). All interviewees consented to the voice recording of the sessions. An overall introduction to EHRs redactable signatures and the eHealth use case scenario was given before the mock-up’s UI testing.

Participants were given an overall task: to log in, sign the EHR of a made-up patient *Josh Brown*, and then export it to the Cloud portal. The latter task given to participants is made up of a sequence of mock-ups pages. The main mock-up pages and the main theme of discussion that corresponds to our research questions are as follows:

1. Sign-in page: 2-factor authentication for authenticating the signer
2. Dashboard page: viewing and selecting EHRs
3. View unsigned Josh Brown’s medical record page: overview and showing possible redaction templates
4. Signing the document: signature visualization

### Group Walk-Throughs: Focus Groups

In our scenario, once a doctor has signed the EHR with a redactable signature, patients will be able to redact their medical documents in the Cloud portal. In this study, the Cloud portal mock-ups were used for addressing the redactors’ point of view, that is, the patients. In our FGs, we had gathered participants who could be those potential patients. FGs allow us to have in-depth discussions with different sets of users and understand their standpoint regarding redactions. The nature of a group encourages discussions and generates interactivity among participants. In addition, in our study design, the first part of our FG sessions included an interactive persona discussion (see below) that required a group discussion and interaction for an in-depth elaboration of the participants’ attitudes and perceptions of selective disclosures.

In addition, we have chosen to involve user groups with different levels of technical expertise and knowledge of cryptographic tools to test whether background knowledge with encryption would influence trusting the validity of the signature after the redaction of medical documents. In particular, we wanted to investigate whether the technical users would expect that a redactable signature rather works similarly as a traditional digital signature and what that would imply in terms of their trust in the system.

### Protocol

The FG sessions lasted approximately 2 hours, including lunch (all but FG5, which lasted 1 hour and 30 min). Consent forms were handed out for participating in the study and for recording the session (see Multimedia Appendix 2. Consent form for focus groups participants); all participants consented to the voice recording of the sessions. Participants were reminded not to disclose any personal information about themselves but rather discuss from the perspectives of the personas, that is, made-up persons that were assigned to them.

The first part of the session included a small exercise that included redactions of personas’ information on papers to understand their perspectives on information privacy and sharing meaning that they were given cards with information describing their personas. A persona consisted of first name, age, weight, marital status, address, hobbies and interests, occupation, salary, medical condition(s), religious affiliation, political affiliation, and sexual orientation.

They were given a few minutes to read their persona’s cards and blackout or redact information they will not disclose to their fellow FG participants. They were instructed to disclose only their personas’ name, whereas the remaining information is optional. Finally, they were asked to present the information they chose to keep and share with their fellow FG members.

A general discussion followed on why some information was not shared by participants and reasoning behind selective disclosure, on the importance of hiding some information, and in which context. We asked participants to stick to the context of a FG. The discussion focused on sharing information in FGs such as the one they are participating in using their personas’ cards.

After the general discussion, an overall introduction to the eHealth use case scenario was given; however, redactable signatures were not described to the nonlay user groups (FG2, FG4, and FG5) but rather later explained after the mock-up’s evaluation was done. One volunteering participant was chosen to control the walk-through of the mock-ups. The main task given to participants is to sign in on behalf of the persona Josh Brown, redact the document, and then send it to the Cloud. The following are the main mock-up UI themes of discussion that corresponds to our research questions:

1. Sign-in page: 2-factor authentication for the redactor
2. Dashboard page: viewing and selecting EHRs
3. EHR redaction: blacking-out metaphor
4. Redaction templates: support and guidance

### Mock-Ups User Interface Designs

Low fidelity mock-ups have been designed using Balsamiq tool for wire framing [26] to clearly signal to the test participants that the discussions should focus on the general functionality and not on specific design issues. On the basis of the requirements and analysis of redactable signatures in Alaqra et al’s study [19], Table 1 shows the list of main HCI requirements that served as a basis for the mock-ups design.

**Table 1:** Redactable signatures’ requirements to mock-ups design.

Requirement index	Description
RQ^a^1	Unobtrusive, easy-to-use, and multifactor authentication
RQ2	Private Cloud run by authorities and branding of (trustworthy) system owner
RQ3	Support (eg, templates) or guidance on redaction considering both privacy and safety
RQ4	Clear responsibilities, that is, the redactor must be accountable
RQ5	User-friendly signature solutions
RQ6	Suitable metaphors and human-computer interaction concepts

^a^RQ refers to a code used for requirement.

Following the eHealth use case scenario mentioned in the Introduction, the mock-ups UIs make up 2 parts: the hospital platform, which is used by medical staff to sign the EHR and a Cloud portal, where patient can view and redact their signed EHR. Requirement 2 (RQ2) is addressed in the UI in the form of considering the hospital’s own trusted platform and a private Cloud portal. The following subsections include the description of the mock-ups UIs designed and highlight the UIs that are considering the requirement. We highlight our investigation purposes in each part that corresponds to the UI functions and/or features.

#### Mock-Ups Interfaces: Hospital Platform for Medical Staff

##### Signing-In and Two-Factor Authentication: Requirement 1 (RQ1)

**Figure 2:**
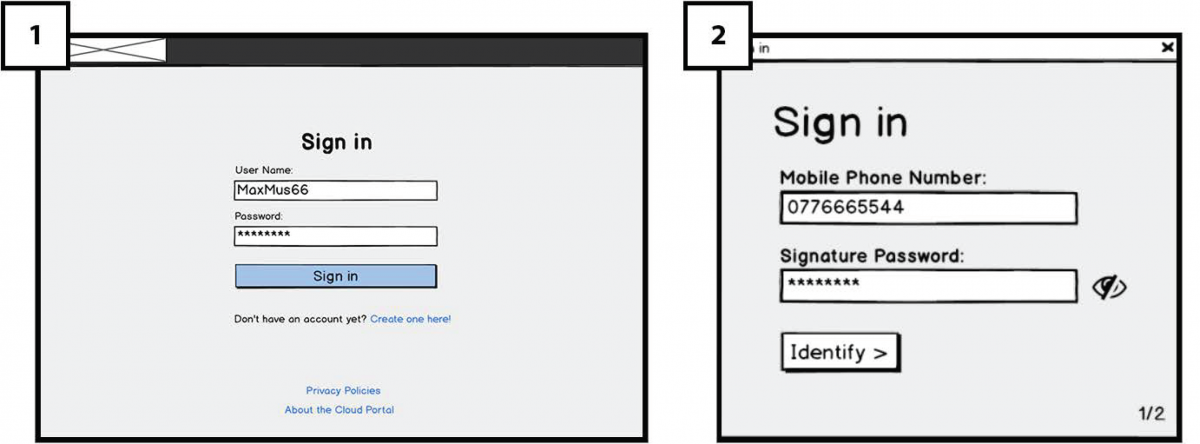
Signing-in and two-factor authentication in Hospital platform using MOXIS.

On the hospital platform’s in Figure 2 (1), *Sign-in* page, the user will enter a user name and a password and then click on the *Sign-in* button, a dialogue box will appear as an extra authentication factor (Figure 2; 2). In accordance with a secure authentication solution of MOXIS’s [27] developed by PRISMACLOUD partner XiTrust and requirement (RQ1), users will use the 2-factor authentication for signing in. They will receive a short messaging service (SMS) text message code on their mobile phone, which is entered into Figure 2 (3) the system before completing the sign-in process. We aimed to test user’s familiarity with the 2-factor authentication process and understand their thoughts regarding its usability and concerns.

##### Dashboard: Viewing and Selecting Electronic Health Records: Requirement 6 (RQ6)

**Figure 3:**
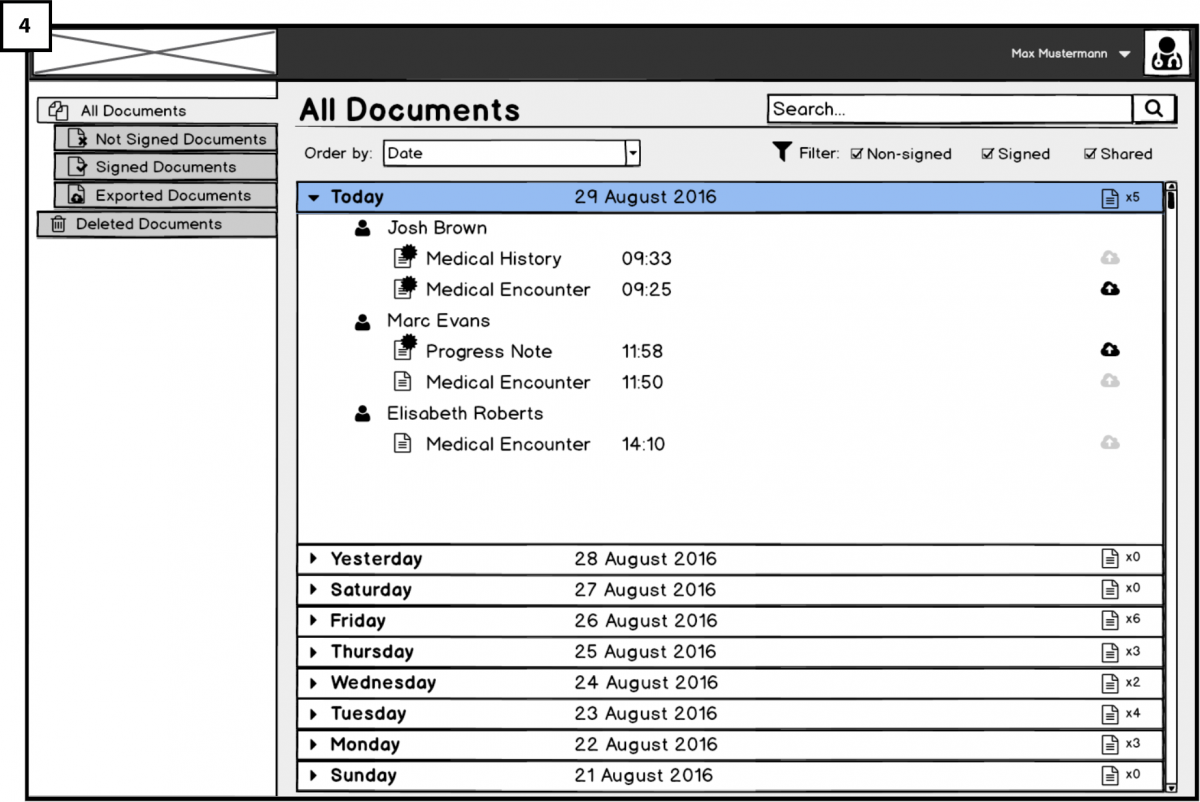
Dashboard of Hospital platform for viewing all electronic health records (EHRs).

Once the doctor has signed in, he or she will reach Figure 3 (4) the home page that shows a list of medical documents and notes produced in conjunction with patient’s encounters. Below the header (in the section’s body), documents are grouped patient wise. Each of these rows includes a document icon, a document title, the time in which the document was created, and a clickable *export to Cloud* icon to the far right.

Above the list, one will find a search field and filtering elements that can be used to search for a particular patient or to filter out nonsigned, signed, and/or shared documents that one is not looking for in the list. We aim to test if the icons are recognizable (ie, if they are suitable metaphors as required by RQ6) and if the documents view matches users’ mental models in real application situations.

##### Overview of Electronic Health Record and Showing Possible Redaction Templates: Requirement 3 (RQ3)

**Figure 4:**
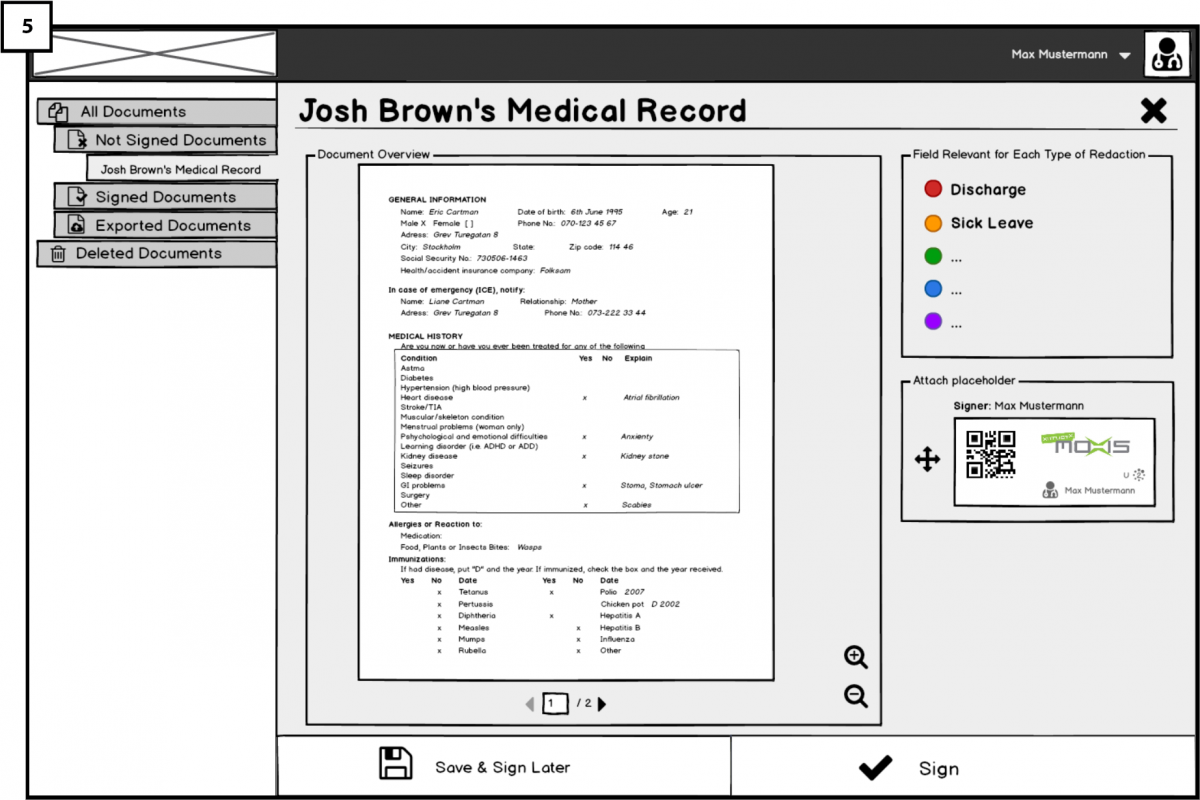
Overview of medical record/electronic health record (EHR) to be signed in Hospital platform.

**Figure 5:**
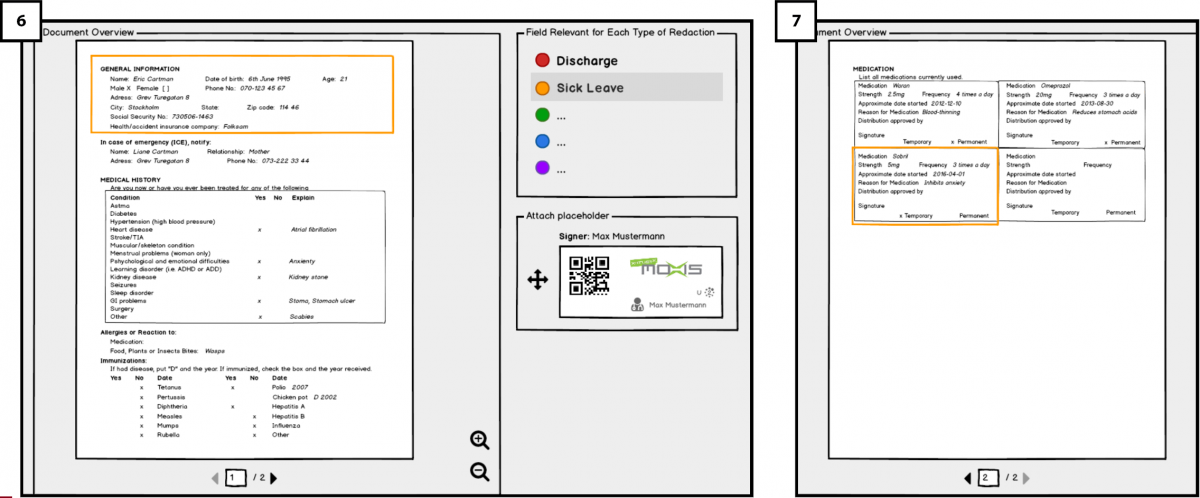
View of possible redaction templates.

When the doctor selects a document to sign, he or she will reach an overview page shown in Figure 4 (5). On the right side of the document overview, the doctor will find a box titled *fields relevant for each type of redaction*, containing different options such as s*ick leave* (allowing patients to conduct redactions for creating a sick leave letter for the employer). These options constitute templates that the patient on the Cloud portal side can use (for different purposes) to create redacted versions of the document, once it has been exported to the patient’s account on the Cloud Portal. The intended use for the display of templates is to guide and show doctors what is meant by redacting documents, opening the room for discussing their opinions regarding redactions and patients redacting their document (RQ3). In addition, we intended to show doctors what might happen if they sign the documents using redactable signatures, documents will remain valid despite some fields being redacting according to redaction rules. In Figure 5 (6), different redaction templates are presented with the possible redactions; highlighted fields correspond to fields remaining after the redactions were done by the patient.

In Figure 5 (7), below the fields relevant for each type of redaction box, there is a signature placeholder that should be attached to the last page of the document before signing it. By attaching the placeholder to the document (through drag-and-drop), the doctor indicates where his or her signature should be placed on the document once it is signed. We are interested to test how the functionality of signature placeholder works and whether users understand it and find it useful.

**Figure 6:**
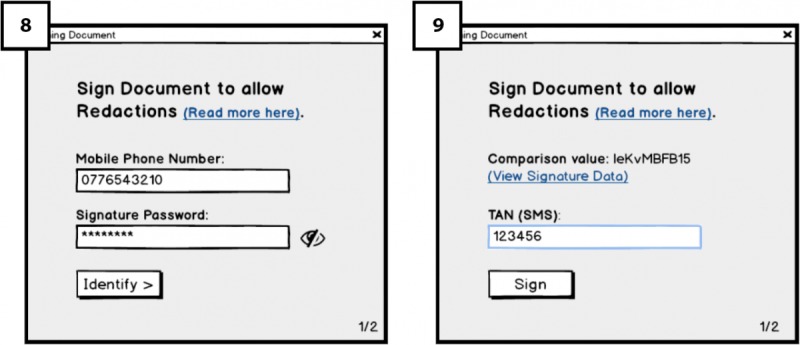
Signing the electronic health record (EHR) with redactable signatures.

After attaching the placeholder and clicking on the sign button, dialogue box (Figure 6; 8) will appear in which the signing is completed through a 2-factor authentication (Figure 6; 9). Thereby, the doctor allows the patient to perform redactions on the document in the future.

##### Signature Visualization: Requirement 5 (RQ5)

**Figure 7:**
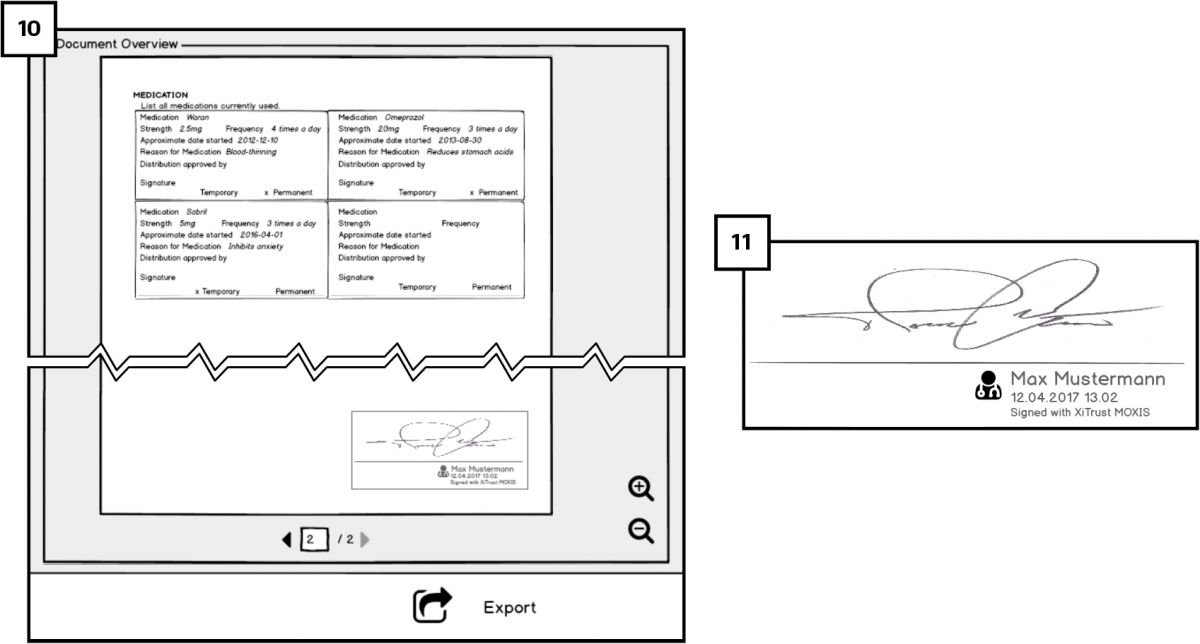
Visualization of the redactable signature and exporting to the Cloud.

The signed EHR with the redactable signature will have a visual representation of the doctor’s handwritten signature. As shown in Figure 7 (10), the handwritten presentation of the redactable signature is shown at bottom of the last page of the EHR. The use of such visual representation of the digital signature (Figure 7; 11) is thought to be more intuitive to users to have a visual confirmation that the document is signed. We aimed to test whether users understand this feature and if it is serving its purpose.

#### Mock-Ups Interfaces Sequences: Cloud Portal for End Users

##### Signing In With Two-Factor Authentication

In similarity to the hospital platform, users will sign into the Cloud portal by using a 2-factor authentication [27].

##### Dashboard: Viewing and Selecting Electronic Health Records

**Figure 8:**
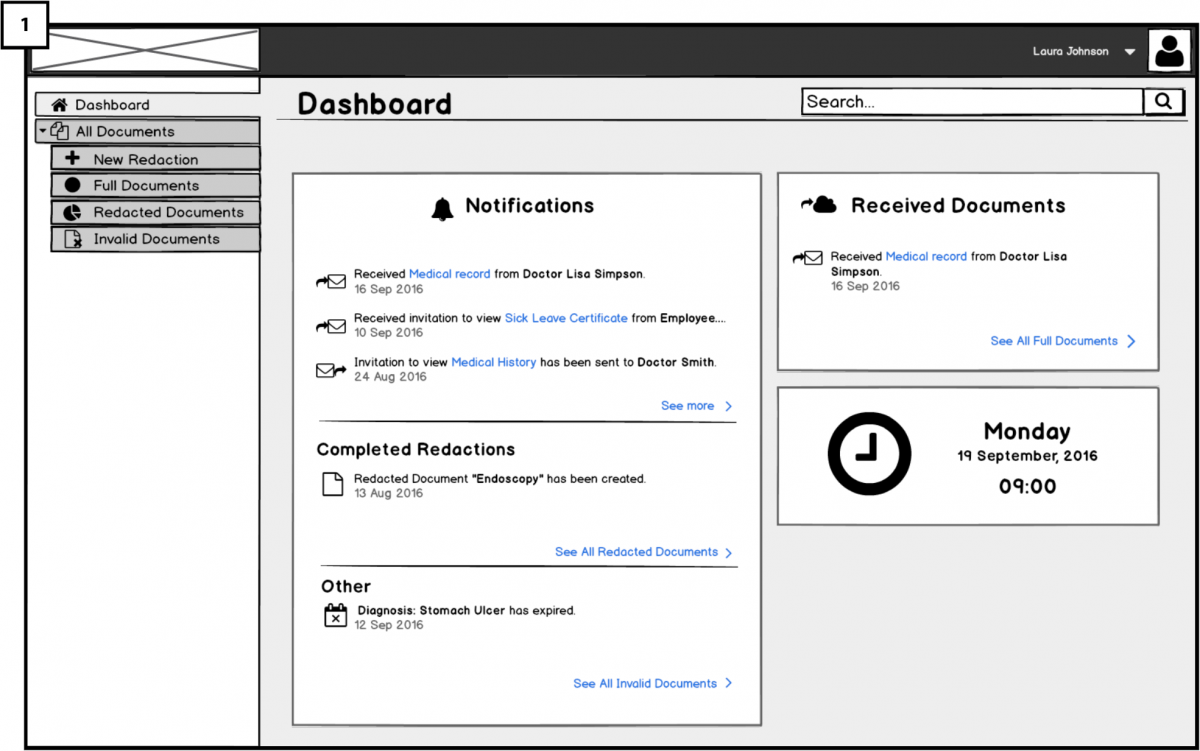
Dashboard of Cloud portal.

After completing the sign-in process, users will reach Figure 8 (1) the dashboard. By clicking on *+ new redaction* in the side menu, users can redact their EHRs.

##### Electronic Health Record Redaction Metaphor: Blacking-Out: Requirement 6 (RQ6)

**Figure 9:**
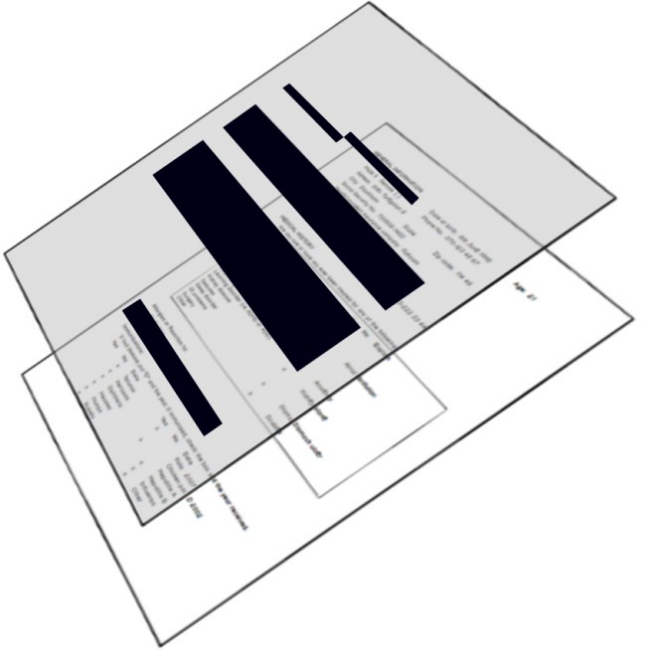
A redaction template metaphor shows the recommended amount of information to redact.

Alternative views on redacted documents for showing either what text will remain and what text will be redacted is based on RQ6. When redacting the EHRs, the metaphor of blacking-out (or more precisely *graying-out*) is used in the form of a *stencil* that is placed on top of the EHR. It is intended to provide patients with guidance on the recommended amount of information to redact from the document (see Figure 9). The text to be redacted is only *grayed-out* with dark gray instead of blacked-out so that patients can still read and check what information will be redacted.

##### Redaction Templates: Support and Guidance Requirement 3 (RQ3)

**Figure 10:**
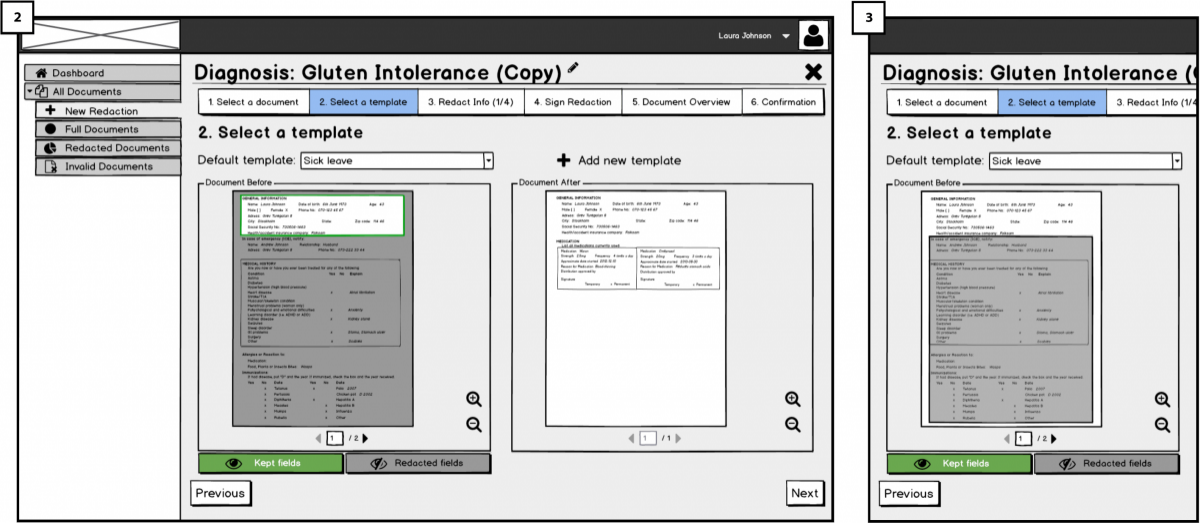
Templates for redaction in Cloud portal.

Our mock-ups UIs address RQ3 by providing a choice of redaction templates that users can use for different contexts, and that should be created by specialists taking both privacy and safety aspects into consideration. In Figure 10 (2), after users have selected a document to redact, they select a template. They can either select a predefined template in a drop-down list (eg, *discharge* or *sick leave*) or create a new template by clicking on the *+ add new template* link on the right side of the list. Below the drop-down list, the template’s effect is indicated in a *document before* and document after view. In the former, users are able to choose between 2 ways of representing redaction: highlight fields that will be kept (Figure 10; 2) or fields that will be redacted (Figure 10; 3). Once the template is selected, users are allowed to redact manually more or less information if they want to, as long as the respective fields are marked as redactable. Patients are redacting information; they should receive immediate visual feedback of the graying out as well as the validity of the doctor’s signature.

##### Signing the Redaction: Accountability of Redaction Requirement (RQ4)

Redactors are requested to perform a keyed-operation when redacting EHRs for making them accountable; thus, addressing RQ4. Therefore, after users have selected the template and/or redactable fields to be redacted, they will need to sign their redaction to complete the process.

**Figure 11:**
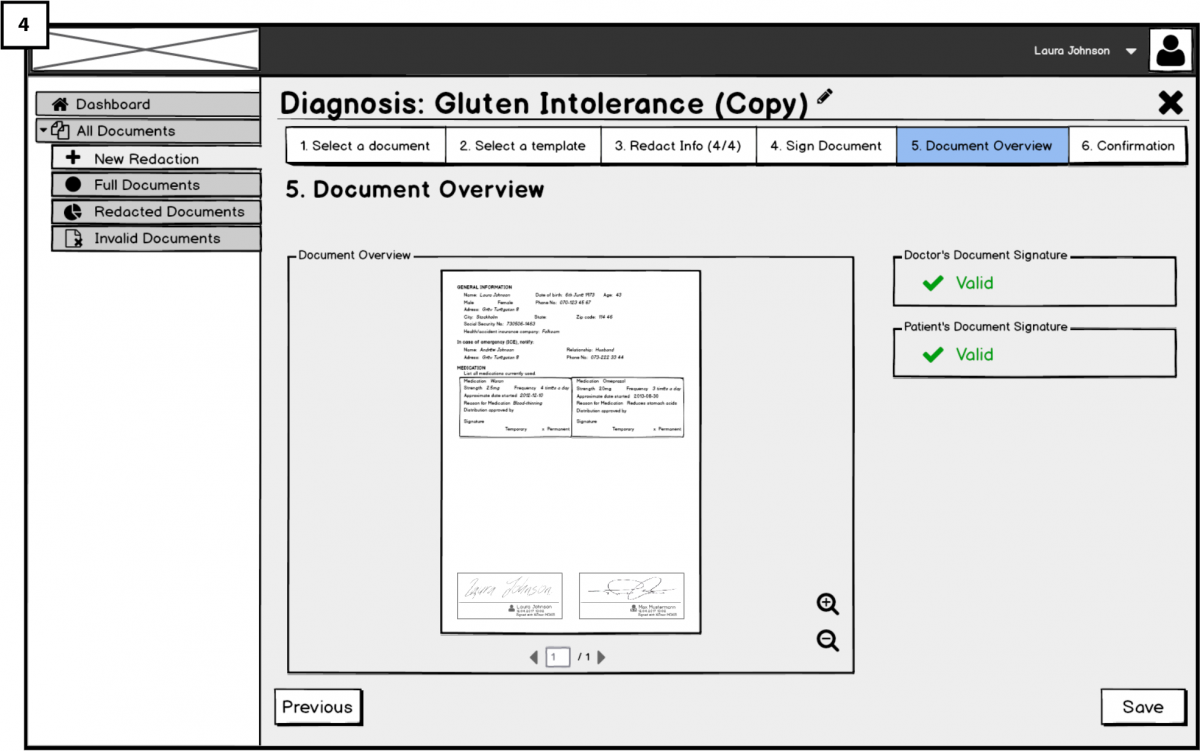
Validity and view of the signature.

This is done by attaching the signature placeholder to the document (similar to the hospital platform) and 2-factor authentication for signing. The final view of the medical document will have both signatures: the doctor’s and the patient’s. Besides, green check icons next to the word *valid* on the right side indicate the validity of both signatures. The validity of doctor’s signature is dependent on the redactions performed by the patient. The patient’s signature next to the doctor’s (Figure 11; 4) is for showing the accountability of the patient who has redacted the document. We aimed to investigate users’ opinions about accountability of redactions and whether the presented solution raises any concerns.

## Results

### Individual Walk-Throughs or Interviews: Medical Staff Perspectives on Signing a Redactable Electronic Health Record

In total, there were 13 interviews, 5 were interviewed in Sweden, Värmland (S1-S5) and 8 in Germany, Frankfurt (G1-G3) and Hamburg (G4-G8). As shown in Table 2, eight participants are doctors in different fields, 2 foundation doctors, 1 nurse, 1 medical secretary, and 1 retired dentist (G4). Most had more than 8 years of experience in their field.

**Table 2:** Overview of medical staff’s working titles and experience.

Index	Working title	Working experience
S1	Medical secretary or care administrator	30 years
S2	Foundation doctor at emergency section	Less than 2 years
S3	Nurse	2 years
S4	Doctor in pathology	20+ years
S5	Foundation doctor in general medicine	Less than 2 years
G1	Dermatologist	20+ years
G2	Doctor and director of cardiology	20+ years
G3	Pediatrician part time psychiatrist	22+ years
G4	Retired dentist	20+ years
G5	Doctor with quality assurance responsibility	20+ years
G6	Doctor of medicine	8 years
G7	General practitioner	25 years
G8	Rehabilitation medical doctor	30+ years

The following sections are the main results from the discussions.

#### Perspectives on Redactable Signatures in Electronic Health Record

Initially, the need for seeing possible redaction templates (as seen in Figure 4), which are made available to the patients as signers for future redaction, was unclear to many participants. However, after a short explanation, most acknowledged that viewing them was important and pointed out concerns regarding the redaction process and the need to consider the following aspects of redaction:

##### Redaction Rules’ Specifications

Many participants mentioned general concerns regarding medical staff having incomplete EHRs, that is, redacted EHR. For instance, G7 emphasized the need for the full document by the recipient and would prefer a discussion with patients before any redactions; however, admitted to lack the time for doing so. Possible misuse scenarios of patients redacting medications to get more drug prescriptions from other doctors were mentioned by S3 and S5.

Many pointed out that there is a need for suitable redaction rules for the patients for conducting their redactions, which restrict the amount of redactable information for maintaining the credibility of the EHR. Suggested rule specifications included that no modifications (beyond redactions) of the EHR should be allowed (S1), the system should be *trusted* (G4 and G6), and that the patient should be the only redactor (G8). S2 and S3 mentioned that redaction rules must be strict, and that in some cases, redactions must not be allowed, for example, in the case of a pilot's medical certificate for heart diseases. The UI should communicate details of the redaction rules to both the doctors and the patients that will follow in addition to the templates.

G2 and G3 expressed strong objection toward patients redacting their medical documents and toward allowing patients to have access control of their EHR. They expressed their distrust in patients' knowledge, expertise, and ability to perform redactions and their distrust of the redacted documents. S4 showed a similar concern regarding the patient's limited knowledge. However, it was regarding patients revealing too much information; while redacting their documents, patients might keep fields that may result in indirect disclosure of sensitive information. Guidance for different types of users should be made clear and minimum data disclosure of the redaction rules should be communicated to the patients.

From these interview results, we can refine our requirements with regard to redaction rules and guidance:

- No arbitrary redactions of EHRs by patients should be allowed.
- Redaction rules are predefined by redaction templates, considering both data minimization and patient safety, in dependence of the type of recipient and purpose of selective authentic EHR exchange.
- Doctors should be able to further fine-tune and restrict the rules for redactions that are made possible to the patients via the templates, that is, the doctors keep the final control of what information is made redactable by the patients via the SAE-service, which they can also discuss and set up in cooperation with their patients.
- Redactions by the patient are restricted by clearly communicated redaction rules, which are given by the templates with possible further restrictions by the doctors.

##### Clear Responsibilities and Accountability

It was noteworthy that all medical staff members have concerns regarding the accountability of the redacted EHR. S4 pointed out repercussions to the doctor as the signer of the redactable EHR and mentioned that the signer might be ‘’sued’’ in some countries for misinterpreted signed redacted document by the patient. Others (S4, S5, and G1) noted that putting trust into an SAE-service would depend on showing that an EHR was redacted and that the redactor is accountable.

Hence, these interview statements helped us confirm requirement RQ4. We derive the following as requirements that:

- Redactions should be enforced by a keyed-operation (ie, confirmed by the patient by a signing operation).
- Redacted EHRs by patients should be clearly communicated to the recipients as having been redacted by the patient, that is, the patient should be made accountable for any redaction. This should be achieved by prominently showing the electronically verifiable signature of the patient for a redacted EHR.

#### Usability of the Authentication and Signing Processes

The Swedish Data Protection Authority *Datainspektionen* has clearly stated, as a rule of thumb, that at least a two-factor authentication mechanism for the processing of sensitive personal data, including medical data, should be used. Although this can directly be derived from the requirement of enforcing appropriate means of security for the processing of personal data pursuant to EU Data Protection Legislation (Art 17 of the EU Data Protection Directive and Art 5 I (f) of the GDPR), our interviews revealed that many clinics in Germany still only use a simple password protection for authentication.

As the redactable signing operation requires a secure authentication of the signer, we also interviewed the medical staff with regard to their perceptions of the 2-factor authentication mechanism of XiTrust’s MOXIS for system log-in and for the signing process (see Figure 2).

##### Efficient Authentication Process

The busy nature of the medical staffs’ working environment requires efficiency for completing their tasks. S5, G2, and G6 raised concerns about the time and efforts consumed, the number of clicks needed to sign-in, and using the mobile phone in our use case, especially when timeouts occur. As S5 stated, “Every micro second counts.” Moreover, 3 participants (S1, S4, and G5), who were familiar with similar 2-factor authentication mechanisms, expressed concerns using SMS for routine work where efficiency is important and thought that it was cumbersome for every instance of signing documents to go through the 2-factor authentication process.

In our studies, participants indicated signing mistakes happening when users were able to sign a document by just a mere click on a sign button. The solution, according to MOXIS developers XiTrust, is intended to eliminate redundant authentication when signing documents. Documents will still be viewed individually, and when reaching the signing process, medical staff can send the approved documents to a *tray* (for bulk signing) where later, they are able to do a group signature by following the authentication process. In this way, they can review documents once again or even have the documents sent to them by other staff (eg, secretaries) to sign. Signing mistakes that have been observed usually happen in the first stage *viewing the document*, and therefore, it is very unlikely in this way of bulk signing, with the extra step to review in the *tray*.

Hence, a multifactor authentication method to be used for secure authentication of the signer should provide efficiency in terms of minimizing the numbers of mouse-clicks required. This could, for instance, be achieved by simply saving username fields and by providing the option to sign a group of EHRs rather than requiring an electronic signature operation for each single EHR.

##### Practically Usable Security

When it comes to signing in, it is clear that medical staff appreciated the added layer of security (2-factor authentication with transaction authentication number(TAN)and short message service (SMS) in comparison with username and password. S1, S4, and G5 were familiar with this 2-factor authentication methods from eBanking apps and had, therefore, no problem in using it.

In addition, some suggested adding 2-factor authentication procedure before uploading medical documents to the Cloud or configuring an extra authentication step. There were, however, practical security concern regarding the use of the SMS and mobile phones for the 2-factor authentication function as expressed by 6 participants (S2, S4, G1, G4, G5, and G7). According to policies of Swedish hospitals, it is not permitted to use personal mobile phones for work purposes; instead, each medical staff is provided with a work phone that is not connected to external networks for security reasons. In addition, some doctors in hospitals in Germany have similar workplace policies for not using smartphones. G5 and G7 mentioned not using it even for personal purposes.

Therefore, requirements for authentication method for the signing operations are as follows:

- The use of commonly known secure authentication solutions that most users are familiar with should be offered (such as Bank-ID in Sweden).
- The UI should offer alternatives for different multifactor authentication methods that do not all require a mobile phone.

##### Human Signing-Error Support

It was reported by some participants (S1 and S4), who already use some signing functionality in their existing systems (however noncryptographic), that mistakes do occur when signing the EHR. Some examples include the hastened clicking on the sign button, especially when multiple parties are involved and discovered errors in the EHR record.

Although our use case requires more steps from the user than a hasty click to sign (authentication process), additional support for medical staff when mistakes occur during the signing of medical documents process is needed. Hence, the functionality of unsigning, that is, revoking a signature of an EHR should be added for mitigating hasty signing actions and for correcting errors.

#### Usability of the Signature Representation

The icon of a *seal* that corresponds to the signed EHR in the mock-ups was clear for most participants; however, S4 noted that a *tick* is more suitable and closer to the real-world analogy.

Most participants were not familiar with digital signatures; therefore, the visual representation of the digital signature was appreciated by them (Figure 7). However, S4, G3, and G8 were familiar with digital signatures. They stated that the visual presentation was not needed and might be ‘’misleading’’ to be the actual digital signature, and therefore, not trusted.

Another concern regarding the visual representation of signatures arises from the case of having multiple parties involved in the signing process: Either multiple doctors or a combination of doctors and medical secretaries, that is, who is to sign first and whose signature is supposed to be there: secretary’s or doctor’s (S4 and G2). Concerns about privacy protection of the doctor were discussed with G5, showing the signature of the doctor on the redacted document is typically revealing the identity of the doctor to the recipient and possibly the doctor may, therefore, be mistaken to have responsibility of the redacted document.

We conclude the following:

- The responsible doctor should add the redactable signature. If the medical secretary should first sign the EHR, this signature could be implemented by noncryptographic means or could later be replaced by the doctor’s redactable signature.
- The roles of the signatures by doctors and patients (as the redactors of EHRs) and the responsibilities of these 2 parties should be made clear by the UI.

#### General Acceptance Criteria

While in Sweden, all EHR are stored electronically available, in Germany, they are mostly stored on paper and not digitalized. Some doctors in Germany (G7 and G8) were hesitating to store very sensitive medical attributes (eg, related to psychiatric diagnoses) electronically or even to upload them to a Cloud platform, as they think that all systems could be hacked, as G8 said, “Hackers are often ahead of things.”

Finally, when asked if they would use the SAE-service to sign a medical document with a redactable signature, S1, S2, and G4 agreed to sign a redactable medical document without further comments. Many participants said yes on the condition of accountability of the patient is clear and redaction is shown (S4, S5, G1, and G5), trusting the system (G6), stricter redaction rules to avoid abuse of drugs (S3), if it is used only for nonmedical uses (G2), and not for all kinds of patients (S5). As mentioned above, S5 and S3 were concerned about drug misuse, for example, a patient hides information about misuse or overconsumption of certain medications (eg, morphine) to get prescriptions from another doctor and suggested some patients to have blocked fields of redaction. G3 was the only participant that stated that he would not sign a redactable medical document as he does not trust the patient’s expertise to redact a document, and therefore, would not trust a redacted document either.

Our interviews showed that acceptance criteria could mostly be met by the refined requirements listed above for clear redaction rules that can be influenced by the doctors and by keeping the patients clearly responsible and accountable. Furthermore, to address any security concerns raised, doctors should have the option to exclude very sensitive fields from the EHR to be signed with their redactable signature and then uploaded to the Cloud platform.

### Group Walk-Throughs or Focus Groups: Patient Perspectives on Redacting Their Electronic Health Record

For addressing patients’ perspectives, we held 5 FGs with a total of 32 participants (Table 3). Out of this, 2 took place in Sweden, 2 in Germany, and 1 in Oslo (at a seminar for information technology [IT] security PhD students in Norway and Sweden).

When recruiting participants, they were asked about their knowledge of digital signatures and redactable (malleable) signatures. Those with none were put in the lay users groups (FG1 and FG3). Those who knew of redactable (malleable) signatures were excluded from the study as our aim was to test the first-hand experience of redactable signatures and test their first thoughts, opinions, and trust they had in the validity of redactable signatures. FG2 consisted of technical users in computing science with the knowledge and experience of digital signatures, whereas FG4 had lay users in executive positions in the industry with knowledge of digital signatures. The fifth group (FG5) consisted of technical experts in the privacy and security field with knowledge and experience of digital signatures (but no knowledge about redactable signatures). The following sections are the main results from the discussions.

#### Users Perspectives on Information Privacy and Sharing

All FGs sessions started with an exercise of redacting personal information fields for different personas on papers (which were assigned and handed to participants). Results of paper redaction exercise are described in Multimedia Appendix 3. Overview results of blacking-out of sensitive data on paper. Almost all participants (30/32, 93%) blacked out information about medical issues. Subsequently, general information such as hobbies, demographics, and address were at the bottom of the chart, where only a few participants (5/32, 15%) redacted them across all groups. There was a clear consensus among participants of all FGs, with no visible cultural differences, regarding the sensitivity of sharing their medical information with fellow FG participants.

**Table 3:** Overview of focus groups participants in each group.

Index	Type of users	Number of participants	Location
FG1	Lay users	6	Sweden
FG2	Technical users	7	Sweden
FG3	Lay users	6	Germany
FG4	Lay^a^ users	6	Germany
FG5	Technical^b^ users	7	Norway

^a^Initially meant to be technical users; however, during the discussion, it was clear that they did not know how digital signatures technically work.

^b^These are security and crypto researchers; their technical expertise goes beyond the other technical users.

The FGs discussions revealed variety of opinions and preferences regarding which information is considered private. Only 1 participant in FG1 expressed not minding sharing all information on the persona’s card, because of personal openness, ideals, and personality trait. In FG3, few participants indicated that the information they would not want to share are reflected by their cultural norms. However, the majority in every group expressed hesitations for sharing most information, especially medical information. In FG2 and FG5, many participants thought they would share more of the information than they already shared voluntarily if they were asked for it, as they do not consider the information to be confidential. In all groups, a prominent factor for sharing information is the context; a couple of participants in FG5 said that they would share different information in medical versus employment environments.

Participants mentioned different contributing factors to sharing more information such as the social environment, depending on persons asking for the information, discussion theme, social norms of the group, society and cultural influences, peer pressure, and social appeal.

As previous research results already showed for the general case [28,29], our evaluations also confirmed that people can be divided into different privacy personas: they have different preferences with regard to withholding personal information, also in dependence on the context of this study.

Hence, we can conclude:

- Different redaction templates offering default redactions should be offered in dependence of the context and type of recipient of the redacted document.
- The UI should motivate the design of the redaction templates for enforcing data minimization by default and protecting patient safety to different types of users.

#### Standardization for Use: Signing-In With Two-Factor Authentication

Most FGs indicated that although the 2-factor authentication is a good idea for security purposes, it is still not clear why they need to use a mobile phone to do so. However, some participants in FG1 and FG2 noted that using 2 different devices is important for a secure signing in as it provides a second secure channel. An alternative suggested by participants in FG1 is to use standard services, that is, the Swedish BANK-ID, which they already use for many other apps. The Bank-ID service is available in the form of a soft certificate that does not require an extra (mobile) device.

These results indicate the need for standardization, requiring to follow a standard format for trust and usability, preferably aligning with existing services or tools by trusted parties. The UI should tailor to secure standard authentication solutions that are commonly used in the corresponding country such as BanK-ID in Sweden.

#### Redaction Rules and Accountability

When discussing redactions in the FGs, the functionality of selective authentic disclosure via redactions was generally appreciated, although there was a clear concern regarding who is responsible for setting up the templates and defining rules of redactions. One participant in FG2 suggested that it should be the recipient as they need to confirm what information they need from the redactor. However, others disagreed based on not trusting the recipient enough to ask for the minimal amount of information (eg, insurance companies might be interested in receiving more information than needed).

The stencil metaphor used for graying out the parts to be redacted was, in general, well understood. However, some participants in FG4 showed concerns in trusting the redaction thinking that the redacted information will still be accessible in a hidden technical manner. It was stated that it is mainly because of their general distrust in programmers that they additionally acknowledged their lack of technical background and knowledge of the system’s processes. Many participants in FG3 and FG4 showed concerns regarding redacted documents as doctors might be still able to acquire the redacted information by other unknown means without the consent of the patient. These concerns were not raised in the FGs with Swedish participants.

Hence, we conclude the following:

- Templates defining redaction rules need to be defined and/or certified by trustworthy actors that are competent to define what information is required considering the data minimization principle and patient safety.
- The fact that redacted data are actually deleted, not simply hidden and unavailable for the recipient, should be clearly communicated by the UI for establishing trust.

#### Redaction and Templates Guidance for Different Types of Users

The stencil metaphor for selective disclosure by blacking out (or in our case, graying out) text to be redacted corresponds to practices in the real world and was well understood by all FG participants. They also acknowledged the desire to disclose selective medical information (redacting EHRs) via redactable signatures. Participants noted that indications showing deviation from the templates were missing from the mock-ups and should be clearly shown in the UI as well as notifications when the signatures become invalid (redaction rules broken) in the process of redacting too much. For example, a warning that their current manual selection for redacting that field is invalidating the doctor’s signature so that they reconsider their actions. The option to select a template for redaction and automatically get a redacted document was mentioned a few times by participants in FG2, where they do not want to further do any redactions and want to shortcut through the process. One participant in FG4 indicated that he or she do not want to choose the template, instead would rather have the system automatically assigning the template based on the recipient.

Users with different background knowledge and experience shared more or less concerns regarding the difficulty of knowing what to include and how much to redact without redacting too much for different recipients. The need for context-dependent templates for enforcing privacy by default while considering patient safety was confirmed. Therefore, the UI should offer templates based on some default recipients for more guidance. Moreover, it has to allow individual adaption of redaction templates. The UI should also show warnings and error messages for redaction rules and diversion from the templates. For users who prefer not to do manual redactions, some default steps with quick redactions following a template selection should be available and incorporated into the UI.

Hence, as concluded above:

- Redactions should be guided by the default templates that the UI should offer in dependence of the recipient.

- In the process of selecting fields to be redacted: when the patient selects fields beyond the permitted redactions, the user should be clearly informed that the doctor’s signature will become invalid.

#### Trust of the Signature’s Validity

For the UI showing the doctor’s document signature as *valid* after the redaction done by the user (Figure 11), we asked all FGs whether they would trust the validity of the doctor’s signature. Evoking the correct mental models for the authenticity of the redacted EHRs and particularly mediating trust in the validity of the redactable signature by the doctor after the redaction had taken place worked for lay users. FGs with lay users (FG1 and FG3) stated that they would have no issues with trusting the signature, participants from FG2 (technical users) were directly questioning the validity of the signature. Some were stating that after changing the text, the signature should be invalid, and 1 participant speculated whether the Cloud portal would create a new signature. In contrast to that, FG5 participants, consisting of experts in privacy and security, were not questioning the validity of the doctor’s signature. We then directly asked them whether they would still think that the signature was valid although the text was changed. Moreover, 1 participant explained that he or she was assuming all redactable fields in the document were signed separately so that those fields with the respective signatures could easily be redacted or deleted without invalidating the validity of the other signed fields, that is, they could see some plausible technical solutions for the validity of the signature.

The FGs showed that depending on the technical knowledge, users might trust or distrust the validity of signatures of redacted documents. Typically, users with some technical knowledge may question the validity, whereas lay users may trust the validity of the signatures and security experts may find technical explanations. Hence, technical and nontechnical users may have different degrees of trust on the validity of the doctor’s signature after redactions.

Therefore, the UI should offer different levels of guidance addressing redactable signatures corresponding to user expertise, that is, introductions or tutorials also have to address technical users and their potential misunderstandings of redactable signatures and the UI should offer tooltip information or a link to explanations what *validity* means for a redactable signature.

#### Branding and Trust

Earlier work has discussed precautions and concerns regarding storing EHR in the Cloud [30] where security, privacy, and trust requirements were stressed.

Concerning branding and trust in the system, some participants from FG2 and FG4 indicated that the UI should clearly indicate which organization is involved. Meaning, which would operate the SAE-service, including the Cloud hospital platform, for example, whether it is the hospital or municipality hosting it and operating a private Cloud. Some participants in FG3 indicated that they would not trust *new technology* that is, the Cloud portal in general; however, many indicated they would trust the governmental authorities and branding of such would be a factor for trusting the system. Some of FG1 and FG2 participants even indicated that they would only trust the authorities in Sweden, whereas others preferred to have options and alternatives. Inversely, 1 participant in FG1 stated that competent privacy or IT security companies, which would often have more skilled personnel than government agencies, are trusted rather than governmental authorities. Nonetheless, FG3 and FG4 participants indicated that there seems to be less trust in the government among the population in Germany.

Hence, based on the above mentioned statement, most participants, especially those from Sweden, seem to trust the government as an operator, some, however, would rather trust competent private IT security companies.

We can conclude overall as follows:

- A trustworthy and creditable agency is needed to brand the SAE-service and to host a private Cloud, considering culturally influenced social trust factors.
- The UI should communicate the trusted party’s branding and privacy or trust certification seals that may be helpful for establishing reliable trust.

## Discussion

### Summary

Main findings of our user studies can be summarized as follows:

In our studies, medical staff’s perspectives on redactable EHRs included concerns regarding redactions resulting in incomplete EHRs used for medical purposes and acknowledgments of patients having control of their own EHRs. Overall, they accepted the Cloud-based SAE-service under the premises of some conditions such as clear responsibilities and accountability of the redactor (patient) as well as rules defining redaction rules. The security of the 2-factor authentication was appreciated, however, required more usable and efficient means for the authentication and signing processes in a hospital environment. In addition, the hand-written signature representation of the digital redactable signature was overall appreciated.

The overall expressed opinions of the FG participants concluded that medical information is most sensitive among other types of personal information such as age, address, and income. Participants well understood the redaction process and the stencil metaphor of *blacking or graying out* of fields in the redaction process. However, they highlighted the need for more support and guidance with regard to redaction templates, for example, different default templates serving different purposes as well as the support for redaction rules. The 2-factor authentication was well received by participants who were familiar with similar apps; thus, standardization with existing solutions was highlighted. Acceptability and trust of the validity of redactable signatures depended on the familiarity and technical experiences of digital signatures. In particular, technically knowledgeable users (apart from crypto-specialists) had had more issues trusting the malleable signatures. In addition, in terms of trust or distrust in the SAE service or in agencies hosting the service, differences existed between FGs in Sweden (higher trust) and Germany (showing distrust).

### Comparison With Previous Work

Addressing privacy concerns and the users’ trust regarding storing their medical data in the Cloud is essential [31-33].

Related work has focused on technical means for addressing privacy and security challenges in EHR systems [34-36]. Studies have addressed patient control in terms of Web-based access to their EHRs for increasing transparency in Sweden [37,38], a dynamic, in terms of defining access control requirements for patients [36], and through a dynamic consent model [39]. Moreover, the acceptability and end-user challenges of personally controlled eHealth records [40,41] have been discussed. However, to the best of our knowledge, no previous work has addressed the end-user perspectives for a Cloud-based SAE-service based on redactable signatures as a means for enhancing patient control over authentic medical data. Consequently, this study is also the first to report about end-user perspectives and requirements for making such service usable, acknowledged, accepted, and trusted by different types of end users.

End-user perspectives for cryptographic selective disclosure technologies, especially in terms of the challenges to evoke comprehensive mental models and to establish end-user trust in the stated selective disclosure functionality have been discussed for attribute-based credentials (ABCs) [15,42] and for the German National identity card [43]. Both ABCs and the German identity card are credentials that provide related user-controlled data minimization functions, which allow the credential holder to selectively disclose attributes or characteristics of those attributes stored on the credentials (eg, they allow to reveal whether a credential holder is over 18 years instead of revealing the exact birth date or any other information stored on the credential). The studies by Wästlund et al and Benenson et al [15,42] explored different ways in which suitable mental models of the data minimization property of ABCs can be evoked on end users. The results showed that while an *adapted card metaphor* helped more than half of the test users to understand that attributes could be selectively disclosed or hidden, nevertheless better design paradigms for understanding the selective disclosure property of attribute characteristics were still needed.

However, redactable signatures used in our use case allow only traditional redactions (ie, deletion of information) as a means for selective disclosure of the remaining information, for which more adequate real-world analogies (such as the *blacking out* metaphor) exist. Blacking out text on paper (including text in letters with signatures) has been long practiced already in the offline world. This may be 1 reason why in this study, the stencil metaphor for blacking out information (illustrated by graying out text in the mockups) worked well for most of our FG participants to understand the selective disclosure property of redactable signatures.

Evoking the correct mental models with our mock-ups for the authenticity of the redacted EHRs and particularly mediating trust in the validity of the redactable signature by the doctor after the redaction had taken place seemed to work well for the lay users. However, technical users that were familiar with traditional digital signatures, which are invalidated by any modifications of the signed text, had doubts in the validity of the signature after the redaction represented by the green *check* icon. These findings are similar to research findings in Lerner et al’s study [44], which report that users with technical security knowledge lacked trust in a newly designed email encryption tools, where cryptographic operations were automatic and hidden, and which, thus, seemed to behave differently to the traditional email encryption tool GNU Privacy Guard (GPG) [45] that they were familiar with. The findings of our study, however, also showed that in the case of redactable signatures, our technical users with advanced expertise in cryptography were also able to find their own technical explanations. Therefore, they were able to establish trust into the validity of the signature by the doctor after the redaction.

### Comparison: Sweden and Germany

Previous studies show that Germany is facing multiple obstacles that prevent the implementation of a national EHR system. Apart from the technical complexity and compatibility issues, there is also a conservatism [20] and strong resistance among health professional organizations against digitalization of health records [22,23]. Moreover, our study showed similar concerns by some of the medical professionals in Germany, who expressed distrust in data security in general or in the patients’ knowledge, expertise, and ability to perform redactions and would, therefore, not trust the redacted documents. The interviewed medical professionals in Sweden did not voice such concerns to that extent.

A higher trust in government agencies for hosting the SAE-service by FG participants in Sweden in comparison with the ones in Germany reflected the Eurobarometer survey results [24], which revealed that people in Sweden are most likely to trust their national public authorities.

Measures providing transparency for the processing of EHRs and accountability of health personnel that are in place in Värmland, Sweden, may also be 1 reason why our FG participants in Sweden, in contrast to the participants in FGs 3 and 4 in Germany, did not voice any doubts that doctors may still be able to obtain the redacted information by other means without the patients’ consent.

In an interview with medical professionals in Sweden, S2 confirmed that accountability measures are taken very seriously in Sweden with the example that doctors require to document the patients’ consent witnessed by a colleague when accessing the patients’ EHRs.

### Legal Rules and Compliance

The proposed SAE-service is compliant with European privacy rules and regulations. It is, in particular, meeting the GDPR’s privacy requirements for data minimization (Art 5 c), and for data protection by design and default (Art 25). It also supports patients to exercise their right to access their medical data by obtaining an electronic copy of their medical records pursuant to Art 15 (3) GDPR and pursuant to national patient data protection legislation (eg, Section 630g (2) Bürgerliches Gesetzbuch in Germany [46] and Section 8 Patient Data Act in Sweden [47]) complementing the GDPR.

Already today, patients would be empowered to redact data from these electronic copies of their medical records (if they are not digitally signed) or from printouts of these copies and pass it on to other parties. The proposed SAE will, in addition, protect the authenticity and integrity of the medial information, and thus, also patient safety, through the (redactable) signature by the doctor. In addition, we require that the patient is digitally signing all redactions (ie, the redaction is implemented as a keyed operation) for making the patient accountable. The secure authentication and signing solution MOXIS by XiTrust that is used for the SAE-service allows to implement the patient’s signature as a qualified signature according to the European Electronic Identification, Authentication, and Trust Services (eIDAS) Regulation with the help of the Austrian trust service provider A-Trust [48]. This means that the patient’s signature would, in this case, fulfill the highest security standards of eIDAS and have the same legal status as a handwritten signature.

### Limitations

As our study’s focus is on the user, our limitations are related to participants of our studies. One might argue that because of our recruitment process (our own network), participants might be inclined to be bias in their feedback. However, because of our research objectives and study design, we refrained from inquiring about their acceptability criteria (if they value our UIs), as that was not an interest for our research. Our focus was on mental perception of the SAE-service functions and sequences, that is, what works and why. Another point is our limited demographic data from our participants. We have had participants varying in background, gender, and age; however, we did not collect that data in our results. In addition, we intended to follow the data minimization principle in practice with our studies: collecting the minimum amount of data necessary for the study.

### Conclusions

In our study, we have addressed medical professionals and patient’s perspectives of our SAE-service. Allowing data minimization of the EHR through redactable signatures, supports users’ control of their medical data. The need for diverse considerations for both roles of users, with different technical backgrounds as well as country they are based in, has been highlighted in our results. One important influence is the effect of users’ experiences on their acceptance and perception of our proposed service that include their mental models and familiarity with existing solutions, experiences with EHRs, and/or their technical background. Therefore, it was challenging in our study to compare user’s acceptance and trust based on countries they are in (Sweden and Germany) as there was a clear distinction in the familiarity and experience of EHRs in the countries addressed. The complexity of different users’ experiences calls for customized designs targeting different sets of users for future usable eHealth solutions.

## Appendix

Multimedia Appendix 1 Consent form for interviews.

Multimedia Appendix 2 Consent form for focus groups.

Multimedia Appendix 3 Overview results: blacking-out of data on paper.

## Abbreviations

ABCs: Attribute-based credentials
eHealth: electronic health
EHR: electronic health record
eIDAS: electronic IDentification, Authentication and trust Services
EU: European Union
FG: focus group
GDPR: General Data Protection Regulation
GPG: GNU Privacy Guard
HCI: human–computer interaction
IT: information technology
ICT: Information and Communication Technology
PbD: privacy by design
PETs: Privacy Enhancing Technologies
PHR: personal health records
PRISMACLOUD: PRivacy and Security MAintaining services in the CLOUD
RQ: requirement
SAE-service: Selective Authentic EHR Exchange service
SMS: short messaging service
TAN: transaction authentication number
UCD: user-centered design
UI: user interface
