# Supplementary figures and images for: Enhancing Privacy Controls for Patients via a Selective Authentic Electronic Health Record Exchange Service: Qualitative Study of Perspectives by Medical Professionals and Patients
Source: J Med Internet Res. 2018 Dec 21;20(12):e10954. doi: 10.2196/10954 (PMC6322916; doi:10.2196/10954)

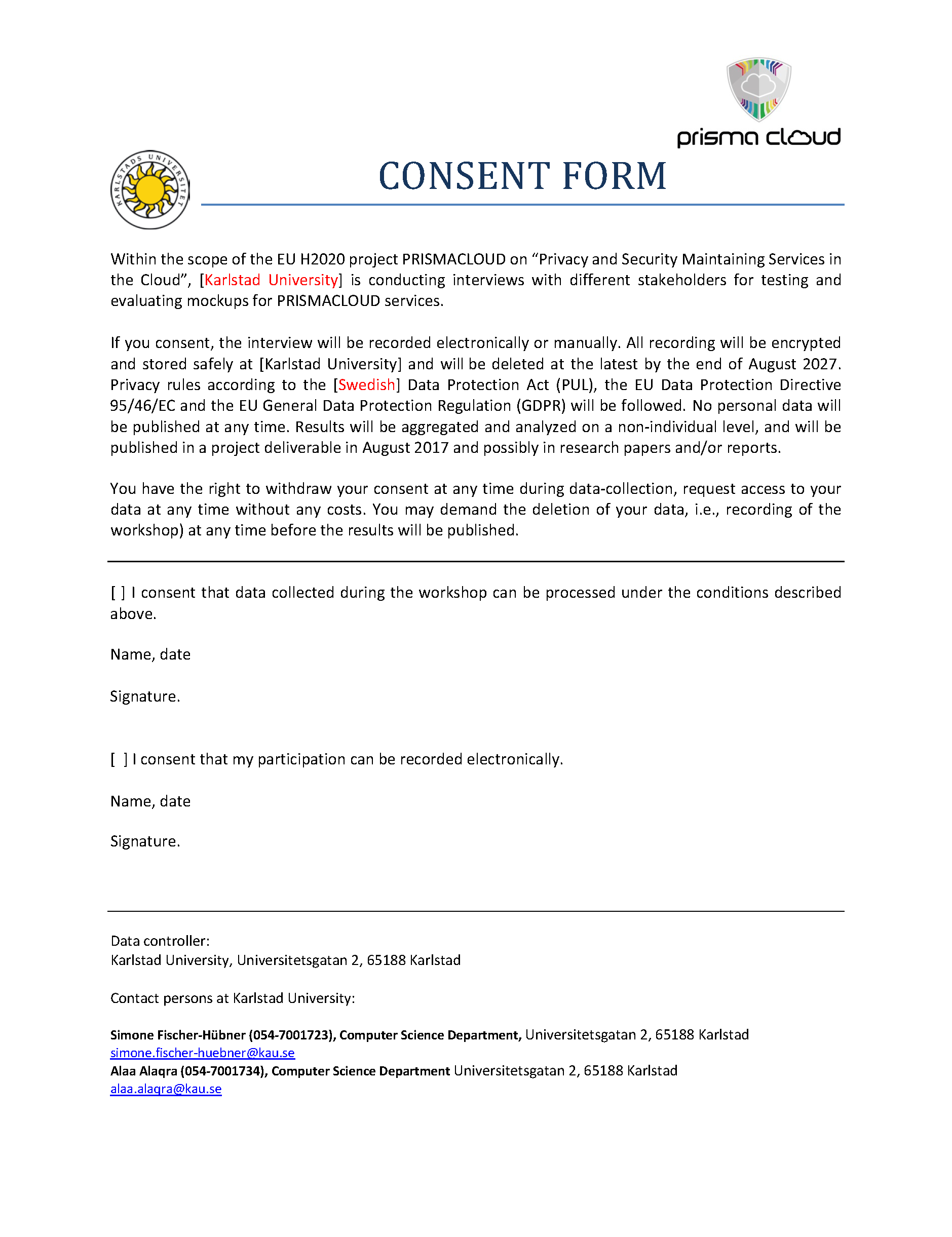

Supplement: Multimedia Appendix 1 [file jmir_v20i12e10954_app1.png]

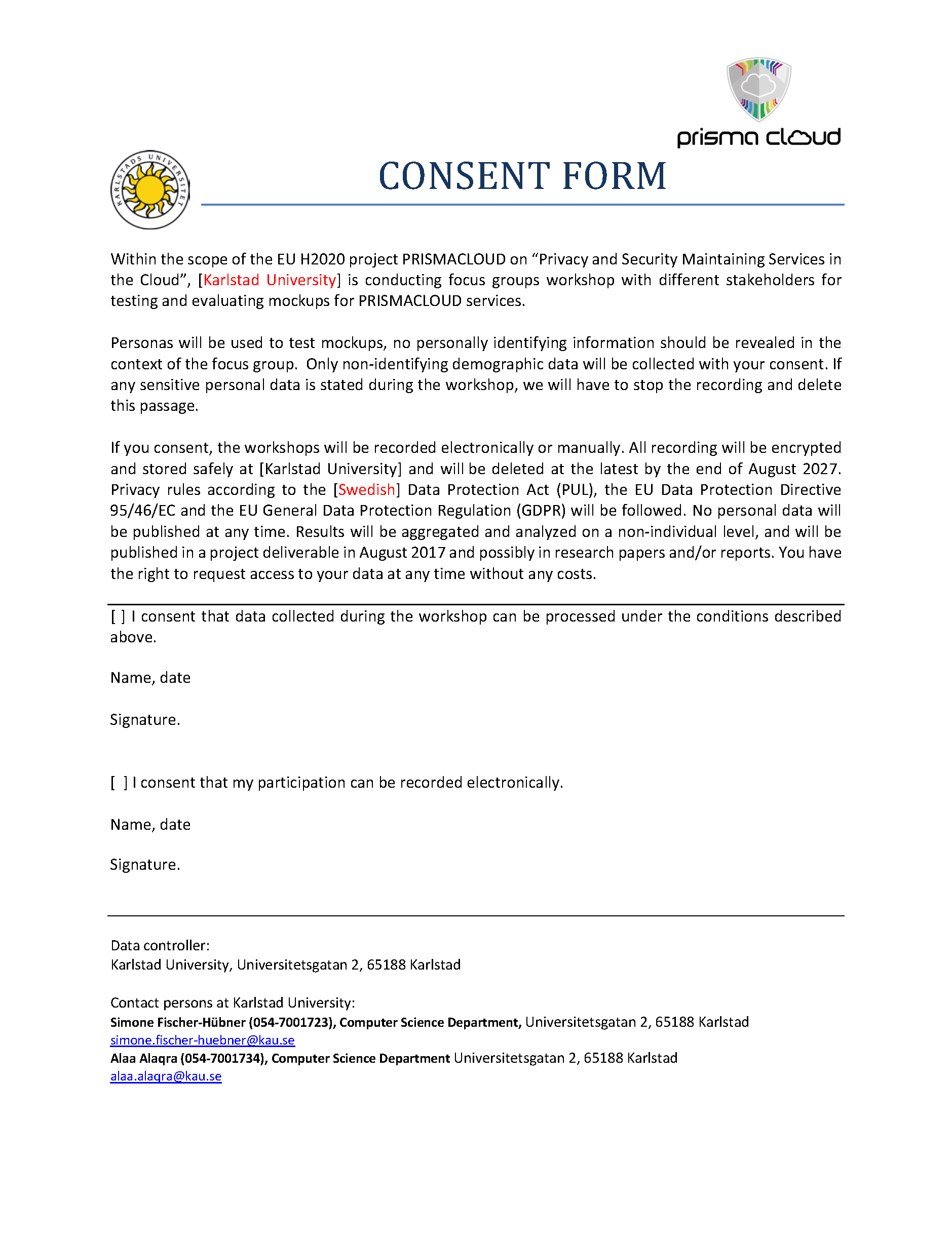

Supplement: Multimedia Appendix 2 [file jmir_v20i12e10954_app2.png]

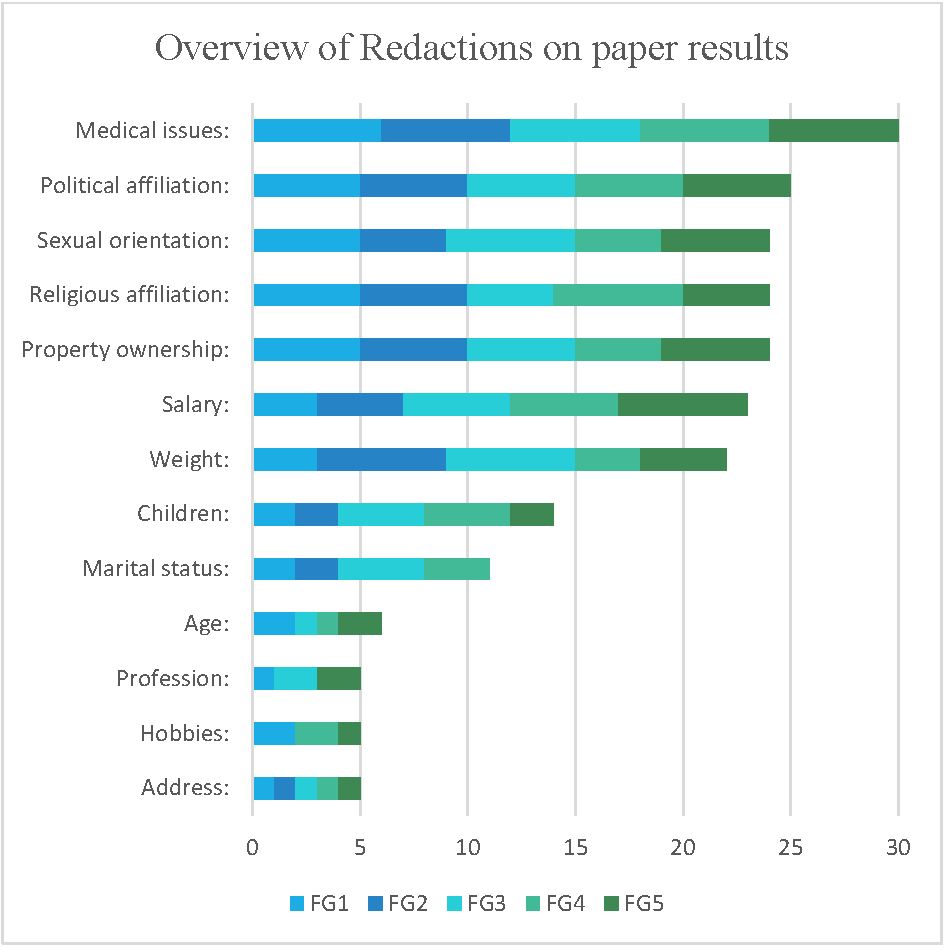

Supplement: Multimedia Appendix 3 [file jmir_v20i12e10954_app3.png]
